# Implementation and reporting of causal mediation analysis in 2015: a systematic review in epidemiological studies

**DOI:** 10.1186/s13104-016-2163-7

**Published:** 2016-07-20

**Authors:** Shao-Hsien Liu, Christine M. Ulbricht, Stavroula A. Chrysanthopoulou, Kate L. Lapane

**Affiliations:** Clinical and Population Health Research Program, Graduate School of Biomedical Sciences, University of Massachusetts Medical School, 368 Plantation Street, Worcester, MA 01655 USA; Division of Epidemiology of Chronic Diseases and Vulnerable Populations, Department of Quantitative Health Sciences, University of Massachusetts Medical School, Worcester, MA 01605 USA; Division of Biostatistics and Health Services Research, Department of Quantitative Health Sciences, University of Massachusetts Medical School, Worcester, MA 01605 USA

**Keywords:** Causal mediation analysis, Systematic review, Causal inference, Causality

## Abstract

**Background:**

Causal mediation analysis is often used to understand the impact of variables along the causal pathway of an occurrence relation. How well studies apply and report the elements of causal mediation analysis remains unknown.

**Methods:**

We systematically reviewed epidemiological studies published in 2015 that employed causal mediation analysis to estimate direct and indirect effects of observed associations between an exposure on an outcome. We identified potential epidemiological studies through conducting a citation search within Web of Science and a keyword search within PubMed. Two reviewers independently screened studies for eligibility. For eligible studies, one reviewer performed data extraction, and a senior epidemiologist confirmed the extracted information. Empirical application and methodological details of the technique were extracted and summarized.

**Results:**

Thirteen studies were eligible for data extraction. While the majority of studies reported and identified the effects of measures, most studies lacked sufficient details on the extent to which identifiability assumptions were satisfied. Although most studies addressed issues of unmeasured confounders either from empirical approaches or sensitivity analyses, the majority did not examine the potential bias arising from the measurement error of the mediator. Some studies allowed for exposure-mediator interaction and only a few presented results from models both with and without interactions. Power calculations were scarce.

**Conclusions:**

Reporting of causal mediation analysis is varied and suboptimal. Given that the application of causal mediation analysis will likely continue to increase, developing standards of reporting of causal mediation analysis in epidemiological research would be prudent.

## Background

Causal mediation analysis identifies potential pathways that could explain observed associations between an exposure and an outcome [1]. This approach also examines how a third intermediate variable, the mediator, is related to the observed exposure-outcome relationship. Causal mediation analysis has been used to study genetic factors in disease causation [2, 3], pathways associated with response to clinical treatments [4], and mechanisms impacting on public health interventions [5, 6]. There are two approaches for conducting causal mediation analysis. The first, primarily applied in the social sciences, involves the comparison between regression models with and without conditioning on the mediator [7]. The second approach uses the counterfactual framework [8, 9], which allows scientists to decompose the total effect into direct and indirect effects [8–13]. Using the counterfactual framework can help to address the potential bias arising from both incorrect statistical analysis and suboptimal study design [14–16].

The field of causal mediation is relatively new and techniques emerge rapidly. With the rapid development of software packages [11–13, 17], the implementation and/or discussion of this methodology is increasing. In a preliminary search in PubMed, we identified 33 articles in 2013, 59 in 2014, and 61 in 2015. While these software packages allow for estimation in a number of settings, limitations on automated procedures for conducting sensitivity analyses on unmeasured confounding or measurement errors remain. However, causal mediation analysis requires careful implementation of the approach and appropriate evaluations for assumptions to derive valid estimates and the extent to which these studies apply and report the elements of causal mediation analysis remains unknown. Therefore, understanding how these methods have been applied to address issues of bias, how studies have implemented the approach, and how estimates are interpreted may provide useful guidance for future reporting.

The purpose of this review was to systematically review epidemiological studies in which causal mediation analysis was used to estimate direct and indirect effects. In this review, we will extract information on the elements critical to be reported and summarize our findings on how epidemiological studies have conducted and presented results from causal mediation analysis. We will also give recommendations for scientists considering to conduct studies applying causal mediation in the medical literature.

## Methods

### Selection of articles

Our aim was to identify original empirical epidemiological research published in 2015 that used causal mediation analysis. Two search strategies were used to achieve this goal. First, we retrieved all published studies citing one of the seminal papers [8, 10, 12, 13] on causal mediation analysis using the Web of Science database. One hundred and fifty-seven articles were identified with this approach. Second, we conducted a keyword search within PubMed through working with a research librarian at the University of Massachusetts Medical School. We developed the following keyword search algorithm: causal mediation analysis OR (“causal” AND “mediation analysis” AND “Mediat*”). This search term returned 61 unique records in PubMed dating from January 1, 2015 to December 31, 2015. We excluded the following types of publications or studies: (i) methodological or simulation studies without an empirical application; (ii) studies without examining the effects on health outcomes, that is, studies not including mortality, morbidity, and diagnostic markers, for both mental and physical health; (iii) animal studies or genetic studies; (iv) letters, meeting abstracts, review articles, and editorials; (v) studies without formal discussion of causal framework or using traditional approach, cross-sectional design, and studies using multilevel models or structural equation models approach.

We used the guidelines from the Preferred Reporting Items for Systematic Reviews and Meta-Analyses [18]. After excluding duplicate records, titles and abstracts of the remaining articles were assigned to two reviewers who independently evaluated each study to assess eligibility. Articles with titles and abstracts were then evaluated by two reviewers through full-text review. Any discrepancy in eligibility was discussed and resolved between reviewers. One reviewer (S-H L) performed data extraction, and two reviewers including a senior epidemiologist (SC and KLL) confirmed the extracted information for all eligible studies.

### Information abstraction

We considered several elements believed to be important for transparent and complete reporting of causal mediation analyses. These included: (1) motivation for applying causal mediation analysis, (2) evaluation of identifiability assumptions of effects identified, (3) use of sensitivity analyses for unmeasured confounding and/or measurement error of mediators, and (4) elements of implementing causal mediations analysis including power calculations, inclusion of exposure-mediator interactions, and bias analysis for interactions. A brief description and rationale for each element chosen is provided in the following sections.

### Rationale for causal mediation analysis

Explanations of cause-effect associations may be enhanced through additional analyses of mediation and interaction. Mediation and interaction phenomena are not mutually exclusive [1]. Several theoretical and practical considerations can also be the motivations to conduct empirical studies for these phenomena of causal effects. Empirically studying mediation can help to: (1) improve understanding; (2) confirm/refute theory; and (3) refine interventions [1]. In this review, we extracted information about whether studies reported (i) the reason for applying causal mediation analysis; (ii) the effect estimates calculated; and (iii) the motivation of the application presented.

### Identification of effects and identifiability assumptions

In a counterfactual framework, three measures are estimated: (1) natural direct effect; (2) natural indirect effect; and (3) controlled direct effect [8, 9]. The natural direct effect expresses how much the outcome (Y) would change if the exposure (A) was set to A = 1 compared to A = 0 (if binary) intervening to set the mediator (M) to what it would have been if exposure had been A = 0 (defined by $\text{Y}_{{1\text{M}}_{0}}-\text{Y}_{{0\text{M}}_{0}}$). The natural indirect effect comparing fixing the mediator to M_1_ versus M_0_ if the exposure is set to level A = 1 (defined by $\text{Y}_{{1\text{M}}_{1}}-\text{Y}_{{1\text{M}}_{0}}$). The controlled direct effect expresses how much the outcome would change on average if the exposure were changed from A = 0 to A = 1 but the mediator were set to a fixed level in the population (defined by Y_1M_–Y_0M_).

For the mediation analysis to have a causal interpretation, we assume that adjustment for the four types of confounding has been addressed. The four types of confounding are: (1) confounding of the exposure-outcome relationship; (2) confounding of the mediator-outcome relationship; (3) confounding of the exposure-mediator association; and (4) mediator-outcome confounders also affected by the exposure [19]. For controlled direct effect, assumptions (1) and (2) are required. For the identification of natural direct and indirect effects, assumptions (3) and (4) are also needed [13]. However, for studies with randomized treatments, assumptions (1) and (3) are satisfied and control only needed to be made for (2) and (4). We extracted information about what identifiability assumptions were acknowledged in relation to identified effects of estimates.

### Sensitivity analysis

In addition to unmeasured confounding common in observational studies [8, 9, 20], measurement error of the mediator could potentially affect the regression coefficient from both the mediator and the outcome regressions and thus result in biased estimates for direct and indirect effects [21–23]. Furthermore, interaction analysis could also be a part of research interests to understand how and why the effect occurs in an observed phenomena. If control has not been made for two sets of confounding factors for each of the exposures, the results from interaction analysis will be biased [1]. In causal mediation analysis, sensitivity analysis can be used as a technique to evaluate the extent to which the direct and indirect effects are robust to assumption violations [24, 25]. We abstracted information on bias analysis to assess: (i) whether sensitivity analysis was conducted or empirically analyzed for identification assumptions; (ii) which identification assumption was a concern and what approach was used for sensitivity analysis; (iii) whether the rationale and approach to conduct sensitivity analysis for measurement errors of the mediators was included; and (iv) whether bias analysis for the interaction was included.

### Power calculations

Studies may be powered to detect a main effect, but may not be sufficiently powered to detect an interaction of a certain magnitude. We hypothesized that many studies implementing causal mediation analyses may be underpowered. We extracted information about power calculations for interaction from each study. However, further development and methodologic work regarding power calculations for direct and indirect effects is needed [1]. With this in mind, we extracted information regarding what authors reported on the issue of power calculations for causal mediation analysis without judgment regarding which formulas were appropriate.

### Exposure-mediator interactions

In the traditional approach for mediation analysis, no interaction between the effects of the exposure and the mediator on the outcome is assumed [8, 9]. Causal mediation analysis, on the other hand, provides the decomposition of the direct and indirect effects that are valid even in the presence of interaction between the exposure and the mediator on the outcome and when non-linear models are needed [8, 9]. This gives rise to the question of when to include or exclude interactions in conducting causal mediation analysis. The decision to include interaction terms is often driven by statistical findings which may be problematic if statistical power is lacking. As such, a recommended approach is to include exposure-mediator interactions in the outcome model by default and only exclude the interaction terms if the magnitude of interactions is small and the estimates of direct and indirect effects are not altered much in the presence of the interaction terms [1]. Leaving the interaction terms in the outcome model is suggested to avoid drawing incorrect causal conclusions, to help allow for additional model flexibility, and to understand the dynamics of mediation [1]. Therefore, we extracted information about whether or not studies allowed for interactions in the outcome model.

### Effects of estimates and results from exposure-mediator interaction

In this review, we assessed whether studies reported both estimates from allowing for exposure-mediator interactions in the outcome model in addition to the effect of estimates without interaction in the model. Moreover, we also extracted estimates from sensitivity analysis conducted for direct/indirect effects and interactions. We also extracted information about explanations of discrepancies when noted.

## Results

**Fig. 1:**
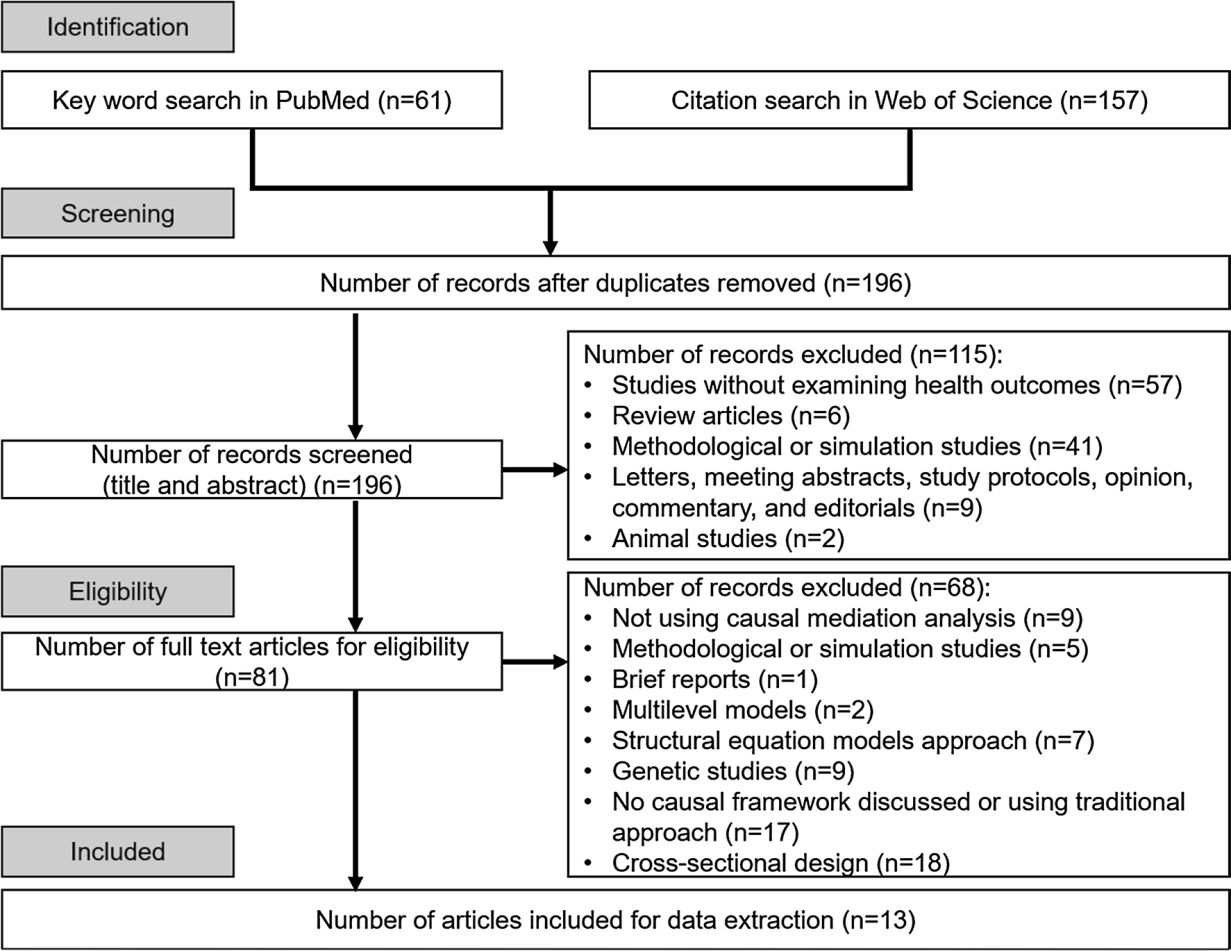
Identification of epidemiological studies using causal mediation analysis in 2015

Figure 1 shows the process of identifying eligible articles for the review. We retrieved 157 and 61 studies from citation search in Web of Science and keyword search in PubMed, respectively. After excluding duplicate studies (n = 22), studies not focusing on the effects of health-related outcomes (n = 57), review articles (n = 6), methodological or simulation studies (n = 46), letters, meeting abstracts and brief reports (n = 10), animals studies (n = 2), studies not using causal mediation analysis (n = 9), genetic studies (n = 9) or studies using multilevel models, structural equation models approach, and cross-sectional design (n = 27), and studies using traditional approach or without formal discussion of formal causal framework (n = 17), we had 13 epidemiological studies that applied causal mediation analysis [26–38].

### Summary of study design, primary exposure, outcomes

Two studies used randomized controlled trials; 8 were cohort studies; and 3 were case–control studies (Table 1). We found that studies were not clustered in one specific area (e.g. 3 studies evaluated risks from environmental exposures including environmental substances [31, 38] and changes in environments [28] and 3 studies evaluated parental conditions before [34, 35] and during [33] pregnancy). Regarding outcomes of interest, 4 studies used the first occurrence of a pre-specified event [32, 35–37]. Other studies also examined levels of biomarkers [27, 30, 31, 38], mortality [26, 29], or neonatal health outcomes [33, 34]. Nearly half of studies used biomarkers as the primary mediator [27, 31, 32, 35, 37, 38]. Other studies used a pre-specified medical event [26, 29, 30], health behaviors [34, 36], psychological symptoms [28], and another a neonatal health outcome [33]. All studies provided information on the confounders in the causal mediation analysis and the majority of studies provided a hypothesized directed acyclic graph (DAG).

**Table 1:** General description of epidemiological studies that were eligible for the systematic review

References	Country, population, and sample size of study^a^	Directed acyclic graph (DAG) included?	Specific mediation hypothesis specified?	Exposure	Primary outcome	Mediator	Confounders^b^
Randomized controlled trials							
D’Amelio et al. [27]	Italy Non-diabetic women with postmenopausal osteoporosis (n = 46)	No	Biologic mechanisms discussed	All treated with calcium 1200 mg/day and cholecalciferol 800 UI/day *Randomized to* with PTH 1–84 100 μg/day subcutaneous Or Without PTH 1–84 100 μg/day subcutaneous (binary)	Glucose metabolism, (continuous, log scale)	Total osteocalcin (OC) undercarboxylated (uOC) (continuous)	Biomarkers that were unbalanced between the two treatment groups at baseline including uOC and serum tartrate resistant acid phosphatase 5B (TRAP5b)
Freeman et al. [28]	England Patients with persecutory delusions from 6 mental health sites (n = 59)	No	Guided by cognitive model of persecutory delusions	*Randomized to* street exposure in areas of relative deprivation during busy mid-day Or A neutral control condition which included sitting in a room watching mildly humorous television clips for 10 min (binary)	(1) State Paranoia using six visual analog scales (VAS) (2) State social paranoia scale (3) Schizotypal Symptoms Inventory—Paranoia (continuous)	*Voices* Hallucinations VAS Distress VAS *Affective* Anxiety VAS Depression VAS Brief core schema scales (BCSS) Self-focus Threat anticipation Interpretation bias (continuous) *Reasoning* *measures* Jumping to conclusions Possibility of being mistaken Alternative explanations Hypothetical contradiction (binary) Probability of being mistaken (continuous)	Baseline measures of paranoia, all of the mediators considered, and center
Cohort studies							
Banack et al. [26]	United States Nationally representative noninstitutionalized Sample of adults aged 20 to 80 years in the U.S. (1988–2004) (n = 7212)	Yes	Guided by previous research	Obesity defined as body mass index ≥30 kg/m^2^ vs. 18.5–29.9 kg/m^2^(binary)	All-cause mortality with follow-up through 2006 (binary)	Self-reported acute cardiac event (e.g. stroke or myocardial infarction) (binary)	Age, gender, race, education, smoking status, and cardiorespiratory fitness
Jackson et al. [29]	New Jersey and Pennsylvania, United States Older adults dually enrolled in medicare and pharmacy assistance programs; “new users” (n = 26,197)	No	Mediators selected based on previous literature	New user of first generation antipsychotic versus new user of second generation antipsychotic (binary)	Mortality with 180 days (binary)	Medical events stroke, ventricular arrhythmia, acute myocardial infarction, venous thromboembolism, pneumonia, bacterial infection (besides pneumonia), and hip fracture) (binary)	70 different demographic characteristics, health service utilization and medication usage, co-existing medical and psychiatric illness, and indicators of functional impairment
Kositsawat et al. [30]	Memphis, Tennessee and Pittsburgh, Pennsylvania, United States Black and white medicare eligible—community dwelling adults aged 70–79 years without diabetes at year 2 of the study (n = 2193)	No	Rationale not clear	Serum vitamin D levels (25-hydroxyvitamin D) <20 ng/mL (binary)	A1c level ≥6.5 % at year 4 (binary)	Diabetes status at year 4 (binary)	Confounders considered in mediation analysis not reported
Louwies et al. [31]	Belgium Working nurses aged between 22 and 59 years without cardiovascular diseases and diabetes (n = 55)	No	Guided by previous literature	Subchronic black carbon exposure (continuous)	Diastolic blood pressure Systolic blood pressure (continuous )	Retinal microcirculation (continuous)	Age, sex, body mass index, smoking, use of anti-hypertensive medication, γ-GT, A1c, distance to major road, clinic, and average weekly temperature
Lu et al. [32]	United States Adults free of coronary heart disease who participated in 9 National Heart, Lung, and Blood Institute funded cohort studies with body mass index ≥ 20 kg/m^2^ (1954–2001) (n = 58,322 for metabolic risk factors; n = 19,572 for fibrinogen analysis)	Yes	Biologic mechanisms discussed	Body mass index (*categories* ≥30 kg/m^2^, 25–<30 kg/m^2^, 20–25 kg/m^2^) (categorical and continuous)	First fatal or non-fatal occurrence of ischemic heart disease, acute myocardial infarction, or angina pectoris (binary)	*Explored in data combined from nine cohort studies* Systolic blood pressure, total serum cholesterol, glucose *Explored in data combined from three cohort studies* Fibrinogen, high-sensitive C-reactive protein (continuous)	Age, sex, smoking, race/ethnicity, socioeconomic status, alcohol intake, physical activity, and dietary intake
Mendola et al. [33]	United States Singleton newborns with ≥23 weeks of gestation (n = 210,610)	Yes	Biologic mechanisms discussed	Preeclampsia (binary)	Ten neonatal outcomes (binary)	Preterm birth (binary)	Study site, maternal age, maternal race/ethnicity, insurance status, marital status, parity, pre-pregnancy body mass index, and chronic diseases during pregnancy
Messerlian et al. [34]	Montreal, Canada Women aging 20–45 years without preexisting medical conditions potentially associated with both infertility and preterm birth and primary analysis was restricted to singleton pregnancies (n = 18,147)	Yes	Noted that the biologic mechanisms are unclear	Reason for infertility (ovulatory, endo-tubal, male factor, uterine abnormalities, unexplained, unspecified) (categorical)	Preterm birth categorized as <32, <35, <37, ≥37 weeks) (ordinal)	Any type of Infertility treatment (binary)	Maternal age, parity, education, smoking, and alcohol or substance use during pregnancy, and body mass index
Raghavan et al. [35]	Framingham, Massachusetts, United States Participants without type 2 diabetes who had whole-genome, common variant genotyping and were followed for a median of 13 years at exam 5 (n = 2361)	Yes	Informed by the literature	Parental history of diabetes—none, one or two parents (ordinal)	Incident type 2 diabetes in offspring (binary)	*Metabolic* corrected insulin response, HOMA-IR, metabolic syndrome, components score *Genetic* genetic risk score *Lifestyle* diabetogenic, diet score, physical activity index (continuous)	Age, sex and genetic risk score (for models not focused on genetic mediators)
Case control studies							
Rao et al. [36]	Karnataka, India Source population from which cases and controls were drawn included adults who were either patients or visitors at 4 major cancer hospitals (n = 452)	Yes	Yes, critical period model guided the DAG construction	Early life socioeconomic disadvantage (low/high)	*Cases* Diagnosed with oral and/or oropharyngeal cancer (ICD-10 codes C00-C10). *Controls* Visitors or those seeking medical care for medical conditions not related to tobacco or alcohol (binary)	Smoking, chewing quid and/or tobacco, alcohol (binary)	Age, sex, adult socioeconomic measures and paternal alcohol drinking
Song et al. [37]	United States Source population from which cases and controls were drawn included postmenopausal women at 40 clinical centers (n = 3049)	Yes	Mediators selected based on previous literature	Low birth weight (ordinal)	*Cases* Self-reported first-time use of medication for diabetes during the follow-up periods *Controls* For each incident case, controls were selected at random from women who remained free from cardiovascular diseases and/or diabetes at the diagnosed time in the case patient (binary)	Biomarkers of insulin resistance, leptin and its receptor, sex steroid hormones and their binding protein, inflammation, endothelial function, cellular ageing and blood pressure (continuous)	Two sets of confounders were considered: (1) Before birth: race/ethnicity and family history of diabetes (2) After birth: age, smoking, alcohol consumption, physical exercise, dietary fiber intake, dietary glycaemic load, and BMI
Xie et al. [38]	Shanghai, China Pre-pubertal and early pre-pubertal boys aged 8-15 years old (n = 167)	No	Yes, biologic mechanisms discussed	Total phthalates (continuous)	*Cases* Diagnosis if constitutional delay of growth and puberty defined by bone age <1.75 years than chronological age *Controls* age and Tanner stage (1 or 2) matched (binary)	Serum testosterone level (continuous)	Age and body mass index

*γ-GT* gamma glutamyl transferase; *HOMA-IR* homeostatic model assessment for insulin resistance; *ICD* international classification of diseases; *PTH* parathyroid hormone

^a^Overall sample size of the study

^b^Confounders included in the causal mediation analysis

^c^The results of mediation analysis were graphically presented

### Motivation for applying causal mediation analysis

The reason for applying causal mediation analysis among all studies was to evaluate mediation (Table 2). With the exception of one study, most studies reported and identified the measures of either direct/indirect effect or controlled direct effect. While the motivation for most studies was to improve understanding, one study used mediation analysis to confirm/refute theory, and one study did not report the motivation.

**Table 2:** Rationale and measures of effect estimated and reported for Causal Mediation Analysis

References	Reason^a^	Measures discussed or reported	Motivation for application^b^
Randomized controlled trials			
D’Amelio et al. [27]	Mediation	Natural direct and natural indirect effects Emphasized direct effect	*Improve understanding* to show that above and beyond how the treatment works through the mediator, there is an independent effect
Freeman et al. [28]	Mediation	Direct and indirect effects^c^ Proportion mediated by various factors	*Improve understanding* of mechanisms
Cohort studies			
Banack et al. [26]	Mediation	Similar to controlled direct effect (with caveat that no manipulation of obesity could actually occur)	*Refute/confirm* that selection bias drives the obesity paradox in cardiovascular disease
Jackson et al. [29]	Mediation	Natural direct and indirect effects Proportion mediated by each medical event	*Improve understanding* of mechanisms
Kositsawat et al. [30]	Mediation	Not identified	*Not clear*
Louwies et al. [31]	Mediation	Direct and indirect effect^c^	*Improve understanding* of mechanisms
Lu et al. [32]	Mediation	Natural direct and natural indirect effect Percent excess risk mediated Natural indirect effect emphasized	*Improve understanding* of mechanisms
Mendola et al. [33]	Mediation	Controlled direct effect	*Improve understanding*
Messerlian et al. [34]	Mediation	Controlled direct effect	*Improve understanding*
Raghavan et al. [35]	Mediation	Direct and indirect effects but only indirect effects reported^c^ Proportion of risk mediated through genetic and metabolic factors	*Improve understanding* of what mediators might be ripe for intervention
Case control studies			
Rao et al. [36]	Mediation	Controlled direct effect	*Improve understanding*
Song et al. [37]	Mediation	Effect not mediated mediated effect^c^ Proportion mediated through various biomarkers	*Improve understanding* of mechanisms
Xie et al. [38]	Mediation	Direct and indirect effect^c^ Proportion of effect mediated through testosterone	*Improve understanding*

^a^Reason for applying causal mediation analysis: Mediation, Interaction, or Interference

^b^Motivation for each application of causal mediation analysis. For *mediation* (1) improve understanding; (2) confirm/refute theory; (3) intervention refinement. For *interaction* (1) help allocate resources better; (2) identifying groups in which treatments may be harmful or beneficial (qualitative or cross-over interactions); (3) understand mechanisms; (4) increase statistical power of main effect analysis, and (5) understand which mediator to intervene upon to eliminate most of the effect of primary exposure. For *interference* (1) quantify spillover effects for cost-effectiveness studies; (2) understand what proportion must be treated to attain population outcomes desired; (3) create knowledge for intervention development and refinement

^c^“Natural” was not specifically used in the article but appeared to have counterfactual framework and appropriate references

### Evaluation of identifiability assumptions and sensitivity analyses

Four studies did not report identification assumptions for measures of effects identified (Table 3). With the exception of two studies, the empirical approach or sensitivity analysis was used to address the issue of confounding. There were 9 studies addressing unmeasured confounding for the mediator-outcome relationship. Five studies provided the empirical approach and four studies used sensitivity analysis to address the concern. For measurement error or misclassification of mediators, 3 studies addressing this issue (Table 4). Two studies provided the rationale for doing sensitivity analysis for measurement error of mediators. Furthermore, they also noted that the bias may result from misclassification of the mediator and robustness of findings was also discussed.

**Table 3:** Examination of Identifiability Assumptions for Causal Mediation Analysis

References	No unmeasured exposure-outcome confounders		No unmeasured mediator-outcome confounders		No unmeasured exposure-mediator confounders		No mediator-outcome confounder affected by the exposure	
	Acknowledged assumption	Empirical analyses or sensitivity analyses	Acknowledged assumption	Empirical analyses or sensitivity analyses	Acknowledged assumption	Empirical analyses or sensitivity analyses	Acknowledged assumption	Empirical analyses or sensitivity analyses
Studies estimating controlled direct effects only								
Banack et al. [26]	✓	Not reported	✓	*Unmeasured confounder* cardiorespiratory-fitness Estimates of the direct effect of cardiorespiratory fitness on mortality from well-established literature. No literature on estimates of prevalence differences of unmeasured confounder—so a range of 10–90 % was considered	Not applicable			
Mendola et al. [33]	✓	Not reported	✓	*Unmeasured confounder* maternal infection Estimates of the direct effect of maternal infection on neonatal outcome ranged from 2 to 10. Prevalence differences of unmeasured confounder—so a range of 1–99 % was considered. Whether this was done because no literature was available on which to base the sensitivity analyses was not reported	Not applicable			
Messerlian et al. [34]	✓	It is unclear if they were addressing this concern although additional pre-specified stratum- specific with different reference categories and exposure groups were used for sensitivity analyses	✓	Stratified analyses “triangulated” those derived from marginal structural models. It is unclear if they were addressing this concern	Not applicable			
Rao et al. [36]	✓	*Unmeasured confounder* situation that unmeasured confounders could be correlated with exposure, mediator, and outcome were considered. Using parameters, such as γ (conditional increase in risk for oral cancer), P1 (prevalence in smokers/chewers/drinkers), and P2 (prevalence among non-smokers/non-chewers/non-drinkers) were specified. The bias introduced by unmeasured confounders that may entirely invalidate the controlled direct effect was calculated	✓	*Unmeasured confounder* considered with the exposure-outcome relationship	Not applicable			
Studies estimating natural direct and indirect effects								
D’Amelio et al. [27]	Randomized controlled trial-not applicable		✓^a^	Not reported	Randomized controlled trial-not applicable		✓^a^	No sensitivity analyses, but adjusted for biomarkers that were unbalanced between the two treatment groups at baseline
Freeman et al. [28]	Randomized controlled trial-not applicable		✓	No sensitivity analyses, but adjusted for baseline confounders; can’t rule out	Randomized controlled trial-not applicable		✓	Not reported
Jackson et al. [29]	✓	Showed risk factors by antipsychotic group	✓	No sensitivity analyses, but adjusted for many risk factors; cannot rule out residual confounding	✓	No sensitivity analysis, but residual confounding (i.e. delirium) at baseline that could bias the total and indirect effects upwards was acknowledged	✓	No sensitivity analyses, but conducted stratified analyses by mediators to provide qualitative evidence for whether or not the association between mediator and mortality is modified by antipsychotic type
Louwies et al. [31]	X	No sensitivity analyses, but adjusted for confounders in Table 1, except day of the week	X	Not reported	X	Not reported	X	Not reported
Lu et al. [32]	✓	Excluded first 3 years of follow-up to reduce the influence of baseline confounders Restricted the analysis to never-smokers to better control for confounding by smoking	✓	*Unmeasured confounder* Common cause of metabolic mediators and coronary heart disease (e.g. family history, genetic factors, residual confounding due to measurement error in diet and physical activity). Sensitivity analyses done with two scenarios: (1) mild confounding (increased hazard ratio by factor of 1.1 and prevalence 20 % for normal weight/25 % for overweight/obese); and (2) strong confounding (increased hazard ratio by factor of 1.8 and prevalence of 45 % for normal weight and 40 % for overweight/obese)	✓	Restricted the analysis to never-smokers to better control for confounding by smoking	✓	Not reported
Raghavan et al. [35]	X	Not reported	X	No sensitivity analyses, but mediation analysis was conducted with all three metabolic mediators (CIR, HOMA-IR and MSS) together	X	No sensitivity analyses, but mediation analysis was conducted with all three metabolic mediators (CIR, HOMA-IR and MSS) together	X	Not reported
Song et al. [37]	✓	No sensitivity analysis, but included all the covariates that may confound the relationship	✓	No sensitivity analysis, but included all the covariates that may confound the relationship	✓	No sensitivity analysis, but included all the covariates that may confound the relationship	✓	Sensitivity analysis was conducted through excluding BMI, a mediator-outcome confounder that is possibly affected by the exposure (low birth weight)
Xie et al. [38]	X	Not reported	X	Not reported	X	Not reported	X	Not reported
Effects not identified								
Kositsawat et al. [30]	X	Not reported	X	Not reported	X	Not reported	X	Not reported

*CIR* beta cell corrected insulin response; *HOMA-IR* homeostatic model assessment for insulin resistance; *MSS* metabolic syndrome score

^a^Identifiability assumptions were not specifically mentioned in the article but appeared to have appropriate references

**Table 4:** Sensitivity analysis for measurement error or misclassification of mediator in causal mediation analysis

References	Mediator	Rationale	Approach	Results
Jackson et al. [29]	Medical events (binary)	Algorithms with high positive-predictive values were used to identify medical events during follow up False negatives is a concern under some scenarios	How results would change were examined given various scenarios of non-differential and differential misclassification Perfect specificity for observing the medical event, but varied the sensitivity from 0.25 to 0.75 separately for those who survived and for those who died was assumed Each scenario was assumed that mediator misclassification was non-differential with respect to antipsychotic type, covariates, and other mediators but some scenarios allowed for differential misclassification with respect to death. A hybrid approach was also used	The proportion mediated was higher than the naïve estimators for some medical events and grew as sensitivity decreased from 0.75 to 0.25. The sensitivity among those who survived, rather than those who died, appeared to have more influence on these results It was suggested that 15 to 45 % of the mortality difference might be explained by some conditions given scenarios assumed compared to 9 % using naïve approach *Authors suggested* to address mediator misclassification when it is suspected, preferably through validation sub-studies or bias analyses
Lu et al. [32]	Biomarkers (continuous)	Not reported	The impact of measurement error in the mediators by calibrating the regression coefficients was assessed Assuming that 1-time measurements for each metabolic risk at baseline explain only 65 % of their true variability (i.e. 35 % measurement error)	After correcting for a presumed 35 % measurement Error in each metabolic risk factor increased the overall the percentage of excess relative risk mediated from 47 % (33–63 %) to 69 % (52–87 %) for overweight, and from 52 % (38–68 %) to 73 % (58–88 %) for obesity
Rao et al. [36]	Smoking Chewing quid and/or tobacco Alcohol (binary)	Dichotomization of mediator variable was done to simplify the analysis but the estimates from the analysis could be biased The sensitivity analysis for non-differential misclassification error of binary mediator was used	The predictive value weighting estimators for outcome regression was used The sensitivity analysis was carried out without accounting for the clustering using the plausible sensitivity values ranging from 0.75 to 1.0 and specificity from 0.75 to 1.0	In the absence of exposure mediator interaction, the sensitivity analysis indicated a slight over estimation of the controlled direct effect The bias seemed to be larger when the sensitivity and specificity decreased

### Elements for implementation of causal mediation analysis

Most studies had a relatively large sample size (Table 5). Three studies had small size (n < 100) and this limitation was acknowledged. The majority of studies did not report whether the power or sample size calculation was calculated. For exposure-mediator interaction, most studies did not report or did not have the exposure-mediator interaction in the model. Among those six studies allowing for exposure-mediator interaction in the model, none reported power or sample size calculation and bias analysis for the interaction.

**Table 5:** Elements of implementation for causal mediation analysis

References	Sample size^a^	Power and sample size calculation for mediation analysis	Exposure-mediator interaction in the model	Power and sample size calculation for interaction analysis	Lack of power mentioned as a non-causal explanation of findings
Studies with negative findings					
Freeman et al. [28]	N = 51 for adjusted and n = 54 for unadjusted analysis	80 % power to detect large indirect effects (Fritz M, Mackinnon DP. Required sample size to detect the mediated effect)	Not reported	Not applicable	Limited power to detect whether mediated effects were statistically significant
Jackson et al. [29]	N = 26,197	Not reported	✓	No	No
Kositsawat et al. [30]	N = 1765	Not reported	Not reported	Not applicable	No
Louwies et al. [31]	N = 55	Not reported	Not reported	Not applicable	Called for cautious interpretation given small sample size
Mendola et al. [33]	Varied by outcomes (i.e. 1 study site did not report infant apnea)	Not reported	✓	No	No
Studies with positive findings					
Banack et al. [26]	N = 7212	Not reported	✓	No	No
D’Amelio et al. [27]	N = 37	80 % power, two-sided significance level of 0.05, to detect differences in uOC greater than 1.71 (t test on log-scale)	✓	No	Acknowledged small sample size as a limitation
Lu et al. [32]	N = 58,322 for metabolic N = 19,572 for fibrinogen	Not reported	✓	No	No
Messerlian et al. [34]	All singleton births (n = 18,147); only first births (n = 8651)	Not reported	Not reported	Not applicable	Limited power mentioned; limited sample size to evaluate spontaneous and induced preterm birth separately
Raghavan et al. [35]	Varied by mediators (n = 2159 for diet score; and n = 2098 for physical activity index)	Not reported	Not reported	Not applicable	Lack of power to examine the association in the mediation framework
Rao et al. [36]	N = 433	Not reported	X	No	No
Song et al. [37]	Varied by mediators (biomarkers)	Not reported	✓	No	Despite some significant mediation by several biomarkers, the sample size may still not be large enough to provide more precise estimates or to detect mediation by other potential factors with smaller mediation effects
Xie et al. [38]	N = 167	Not reported	X	No	Small sample size acknowledged

^a^Smallest sample size used in the causal mediation analysis

### Effects of estimates and derived results from exposure-mediator interaction

Table 6 shows the estimates from causal mediation analysis with and without interaction in the model for the associations between the primary study exposure and outcome listed in Table 1. While the majority of the studies reported estimates from either with and or without interaction in the model, 3 studies did not report identified estimates of effects. Among 6 studies allowing for exposure-mediator interaction, 2 studies presented results from both with and without interaction in the model and no substantial discrepancies were found.

**Table 6:** Estimates of Direct and Indirect Effects With and/or Without Mediator-outcome Interaction

References	Without exposure-mediator interaction (95 % confidence interval)	With exposure-mediator interaction (95 % confidence interval)	Discrepancy found with and without exposure-mediator interaction reason discussed
Banack et al. [26]	Not reported	*Controlled direct effect* with CVD *Risk* *ratio*: 0.62 (0.49, 0.78) *Risk* *difference*: −0.12 (−0.20, −0.04) *Controlled direct effect* without CVD *Risk* *ratio*: 1.30 (1.13, 1.49) *Risk* *difference*: 0.03 (0.01, 0.05) *Total effect* *Risk* *ratio*: 1.24 (1.11, 1.39) *Risk* *difference*: 0.03 (0.02, 0.05)	Not applicable
D’Amelio et al. [27]	Effects of treatment on glucose level at 12 months mediated by OC at 6 months: *Natural* *direct* *effect*: −0.033 (−0.186, 0.121) *Natural* *indirect* e*f*fect: −0.050 (−0.178, 0.078) *Total* *effect*: −0.082 (−0.174, 0.009)	Not reported	Not applicable
Freeman et al. [28]	Anxiety (Boot SE), *P* value *Direct effect*: 0.21 (0.16), 0.19 *Indirect effect*: 0.18 (0.11), 0.09 *Total effect*: 0.39 (0.16), 0.01 Depression (Boot SE), *P* value *Direct effect*: 0.24 (0.14), 0.09 *Indirect effect*: 0.15 (0.11), 0.18 *Total effect*: 0.39 (0.16), 0.01 BCSS—negative self (Boot SE), *P* value *Direct effect*: 0.33 (0.18), 0.06 *Indirect effect*: 0.06 (0.08), 0.48 *Total effect*: 0.39 (0.16), 0.01 BCSS—positive self (Boot SE), *P* value *Direct effect*: 0.40 (0.17), 0.02 *Indirect effect*: −0.01 (0.05), 0.92 *Total effect*: 0.39 (0.16), 0.01 BCSS—negative other (Boot SE), *P* value *Direct effect:* 0.22 (0.16), 0.17 *Indirect effect*: 0.18 (0.11), 0.13 *Total* *effect*: 0.39 (0.16), 0.01	Not applicable	Not applicable
Jackson et al. [29]	*Stroke* *Direct* *effect*: 1.13 (1.05,1.22) *Indirect* *effect*: 1.005 (1.001,1.011) *Total* *effect*: 1.14 (1.06,1.22)	*Stroke* *Direct* *effect*: 1.13 (1.06,1.22) *Indirect* *effect*: 1.005 (1.001,1.011) *Total* *effect*: 1.14 (1.06,1.22)	No Not applicable
Kositsawat et al. [30]	Not reported	Not applicable	Not applicable
Louwies et al. [31]	Systolic blood pressure *Direct* *effect*: 2.93 (CIs not reported) *Indirect* *effect*: −0.42 (−1.35 to 0.17) *Total* *effect*: not reported Diastolic blood pressure *Direct* *effect*: 3.15 (CIs not reported) *Indirect* *effect*: −0.59 (−1.44 to 0.07) *Total* *effect*: not reported	Not applicable	Not applicable
Lu et al. [32]	*Overweight (for* *metabolic mediators)* Blood pressure *Natural* *direct* *effect*: 1.16 (1.09–1.24) *Natural* *indirect* *effect*: 1.06 (1.03–1.08) *Total* *effect*: 1.22 (1.14–1.30) *Obesity (for* *metabolic mediators)* Blood pressure *Natural* *direct* *effect*: 1.28 (1.15–1.43) *Natural* *indirect* *effect*: 1.13 (1.07–1.19) *Total* *effect*: 1.42 (1.25–1.60)	*Overweight (for* *metabolic mediators)* Blood pressure *Natural* *direct* *effect*: 1.16 (1.09–1.24) *Natural* *indirect* *effect*: 1.05 (1.02–1.08) *Total* *effect*: 1.22 (1.14–1.30) *Obesity (for* *metabolic mediators)* Blood pressure *Natural* *direct* *effect*: 1.28 (1.15–1.42) *Natural* *indirect* *effect*: 1.10 (1.03–1.17) *Total* *effect*: 1.43 (1.25–1.62)	No Not applicable
Mendola et al. [33]	Not reported	*Peri*- *or intraventricular hemorrhage:* Controlled direct effect: 3.2 (1.4–7.7) *Total* *effect*: 2.9 (2.4–3.4)	Not applicable
Messerlian et al. [34]	*Uterine:* <*35* *weeks* Controlled direct effect: 2.43 (0.85, 6.93) Total effect: 2.27 (1.32, 3.89)	Not applicable	Not applicable
Raghavan et al. [35]	*Metabolic syndrome score:* Direct effect: not reported Indirect effect: 1.20 (1.07, 1.33) Total effect: not reported	Not applicable	Not applicable
Rao et al. [36]	*Smoking: low* vs*. high* Controlled direct effect: 1.5 (1.4, 1.5) Total effect: 1.6 (1.4, 1.9)	Not applicable	Not applicable
Song et al. [37]	HOMA-IR: *Effect* *not* *mediated*: 1.32 (0.95, 1.88) *Mediated* *effect*: 1.22 (1.02, 1.49) SHBG: *Effect* *not* *mediated*: 1.97 (1.25, 3.10) *Mediated* *effect*: 1.16 (1.03, 1.33) E-selectin: *Effect not mediated*: 1.56 (1.10, 2.21) *Mediated* *effect*: 1.12 (1.02, 1.23) Systolic blood pressure: *Effect* *not* *mediated*: 1.61 (1.25, 2.16) *Mediated* *effect*: 1.03 (1.01, 1.07)	Not reported^a^	Not applicable
Xie et al. [38]	Not reported	Not applicable	Not applicable

*BCSS* brief core schema scales; *CI* confidence interval; *CVD* cardiovascular disease; *HOMA-IR* homeostatic model assessment for insulin resistance; *OC* osteocalcin; *SE* standard errors; *SHBG* sex hormone-binding globulin

^a^Despite allowing for interaction, only models assuming no interaction were adopted due to no significant interaction between any of the exposures and mediators was observed

## Discussion

Our review shows that reporting of research on methods using causal mediation analysis to better understand mechanisms of observed exposure-outcome relationship is varied and suboptimal in the field of epidemiology. After reviewing 13 epidemiological studies, we found that while the field of causal mediation analysis has made significant strides, majority of the studies lacked sufficient details on whether the identifiability assumptions were satisfied in relation to identified effect estimates. Furthermore, despite most studies addressing the concern for unmeasured confounders either from empirical approaches or sensitivity analyses, we found that over half of studies did not examine the potential bias arising from the validity of the mediator. In addition, the majority of studies did not provide or comment information on the power calculation or issues of sample size. While some studies allowed for exposure-mediator interaction, only a few presented results from both with and without interaction in the model.

Although it was difficult to judge the adequacy of control for confounding in the reviewed studies without increased knowledge of the specific datasets and subject areas, we found that most studies did not provide enough information on whether either the empirical approach or sensitivity analysis was conducted for identification assumptions in relation to effect estimates identified. It has been emphasized that controlling for mediator-outcome confounders is important when direct and indirect effects are examined [8, 9, 20]. When there is concern for uncontrolled confounding, sensitivity analyses have been recommended to quantify the extent to which the unmeasured confounding variable would have to be to invalidate inferences about the direct and indirect effects [15, 24, 39]. Several approaches can be used to address unmeasured confounding [1]. For example, researchers can choose to report how large the effects of the confounder variable would need to be to completely explain the effects of estimates. To improve reporting of causal mediation analyses in epidemiological literature, we recommend the following. First, studies should be transparent on whether the empirical approach or sensitivity analyses were used to evaluate identifiability assumptions. Second, studies must carefully consider the extent to which bias is present due to concerns regarding valid measurement of the mediator. Several approaches are available to address this issue [21, 22]. Third, if researchers are concerned about the presence of multiple bias in the study, we recommend that researchers prioritize the approaches depending on the context to strengthen their findings.

We found that the majority of studies did not report whether the statistical power or sample size calculation was calculated or if the researchers believed that the sample size available was sufficient to estimate direct and indirect effects with sufficient precision. However, we recognize that approached for calculation power and sample size for direct and indirect effects is limited in the current literature, especially for the exposure-mediator interaction [1]. To understand what sample size is sufficient for mediation analysis, it is currently recommended that researchers use previously published tables for adequate power in single-mediator models [40]. In addition, we also recommend that studies should comment on whether lack of power or insufficient sample size was a likely non-causal explanation of findings especially for these with relatively small sample size.

It has been proven that under sequential ignorability and the additional no-interaction assumption, the estimate based on the product of coefficients method can be interpreted as a valid estimate of the causal mediation effect as long as the linearity assumption holds [41, 42]. However, in many studies it is unrealistic to assume that the exposure and mediator do not interact in their effects on the outcome. Carrying out mediation analysis incorrectly assuming no interaction may result in invalid inferences [13]. Despite the progress of statistical methods in mediation analysis under settings with a binary mediator or count outcomes for exposure-mediator interactions [13], we found that most studies did not report whether there was exposure-mediator interaction in the model.. Although more assumptions are required for the decomposition of a total effect into direct and indirect effects even in models with interactions and non-linearity under the counterfactual framework, this decomposition of total effects allows investigators to assess whether most of the effect is mediated through a particular intermediate or the extent to which it is through other pathways. Therefore, we recommend that future studies include exposure-mediator interactions by default in the outcome model as suggested [1]. We recommend that exposure-mediator interactions only be excluded if the magnitude of interactions is small and do not change the estimates of direct and indirect effects very much.

Our review is subject to some limitations. First, we included only epidemiological studies published in 2015 and limited to those published in English. The findings may not be representative of all publications using causal mediation analysis. However, it is reasonable to give some time for the development and use of methods given that seminal articles for applications were mainly published in 2012 or 2013 and we are interested in a “snapshot” of current practices in reporting such complex methods from the most recent year. Second, the reporting practices of published studies may be influenced by journals’ requirements. Authors may be reporting their approach and findings given word limitations from journals and thus may have limited space to provide details needed for the method. Nevertheless, with methods that require careful implementation of the approach, such reporting is necessary to evaluate the extent to which the method has been appropriately applied. Third, it is possible that we missed some relevant articles due to lack of standardized terminology or exchangeable jargons to describe the study design of causal mediation analysis. However, we believe that including papers which cited the seminal papers reduced the likelihood of this happening. Despite the limitations, this is the first review to examine how epidemiological studies have used causal mediation analysis, what appropriate procedures and analysis are needed to conduct such complex technique, and what elements are critical to report for the method, which is we believe is a strength of our review.

## Conclusions

Although the application of causal mediation analysis is increasing in epidemiology, there is an opportunity for improving the quality and presentation of this methodology. We found that there is varied and suboptimal reporting of this emerging approach in literature. We identified that the majority of studies addressing unmeasured confounding for the mediator-outcome relationship. We recommend that future studies should: (1) provide sufficient details on whether either the empirical approach or sensitivity analysis was conducted for identifiability assumptions in relation to effect estimates identified, (2) comment on the bias that may arise from the validity of mediator, (3) discuss whether lack of statistical power or insufficient sample size issue was likely a non-causal explanation of findings, and (4) allow the inclusion of exposure-mediator interaction in the model and present results derived from models with and without interaction terms. We hope that the development of best practices in reporting complex methods in epidemiological research and the adoption of such reporting standards may help quality assessment and interpretation of studies using causal mediation analysis.
